# The Role of Thoracic Ultrasound for Diagnosis of Diseases of the Chest Wall, the Mediastinum, and the Diaphragm—Narrative Review and Pictorial Essay

**DOI:** 10.3390/diagnostics13040767

**Published:** 2023-02-17

**Authors:** Ehsan Safai Zadeh, Christian Görg, Helmut Prosch, Rudolf Horn, Christian Jenssen, Christoph Frank Dietrich

**Affiliations:** 1 Gastroenterology, Endocrinology, Metabolism and Clinical Infectiology, Interdisciplinary Center of Ultrasound Diagnostics, University Hospital Giessen and Marburg, Philipps University Marburg, Baldingerstraße, 35033 Marburg, Germany; 2 Department of Biomedical Imaging and Image-Guided Therapy, Medical University of Vienna, Vienna General Hospital, 1090 Vienna, Austria; 3 Center da Sandà Val Müstair, 7536 Sta. Maria, Switzerland; 4 Medical Department, Krankenhaus Maerkisch-Oderland, 15344 Strausberg, Germany; 5 Brandenburg Institute of Clinical Ultrasound, Medical University Brandenburg, 16816 Neuruppin, Germany; 6 Department Allgemeine Innere Medizin (DAIM), Kliniken Hirslanden Bern, Beau Site, Salem und Permanence, 3018 Bern, Switzerland

**Keywords:** ultrasound, ultrasound-guided biopsy, chest wall, diaphragm, mediastinum, cancer

## Abstract

The diagnostic capabilities of ultrasound extend far beyond the evaluation of the pleural space and lungs. Sonographic evaluation of the chest wall is a classic extension of the clinical examination of visible, palpable, or dolent findings. Unclear mass lesions of the chest wall can be differentiated accurately and with low risk by additional techniques such as color Doppler imaging, contrast-enhanced ultrasound, and, in particular, ultrasound-guided biopsy. For imaging of mediastinal pathologies, ultrasound has only a complementary function but is valuable for guidance of percutaneous biopsies of malignant masses. In emergency medicine, ultrasound can verify and support correct positioning of endotracheal tubes. Diaphragmatic ultrasound benefits from the real-time nature of sonographic imaging and is becoming increasingly important for the assessment of diaphragmatic function in long-term ventilated patients. The clinical role of thoracic ultrasound is reviewed in a combination of narrative review and pictorial essay.

## 1. Chest Wall

### 1.1. General Examination Technique

For the ultrasound (US) evaluation of the chest wall, a distinction must be made between superficial and deep pathology. Due to their superficial location, pathology of the chest wall is usually examined with a small-part linear transducer with a frequency range of approximately 7–12 MHz. In this regard, the transducer can be considered to be an extension of the examination of a visible pathology, a palpation finding, and, in some cases, of localized pain. The ultrasound section planes should be selected according to the pathological findings. In the near-field region of the chest wall, pathology of the skin, subcutaneous adipose tissue, chest wall musculature, ribs, endothoracic fascia, and parietal pleura can be assigned. Knowledge of sonoanatomy of the chest wall with respect to the course of the vessels (mammary arteries, intercostal arteries), bony ribs, and cartilaginous ribs is required. During the examination of ribs, it should be noted that they run primarily horizontally in the dorsal arch part, steeply caudally in the lateral arch part, and then mostly horizontally again near the sternum (ribs 1–6) or run steeply cranially (ribs 7–10). In the differentiation of pulmonary pathology, attention should be given to the respiratory motion of the lungs [1]. Although the soft tissues of the chest wall can generally be examined well on US due to their superficial position, only the surface reflex with dorsal sound extinction can be visualized from healthy bones. However, interruptions of the cortical bone—whether caused by a fracture or by metastases or primary tumors—can usually be visualized well sonographically. In contrast, the sound waves can penetrate the non-calcified rib cartilage (in the ventral arch part of the ribs), and therefore these areas can be examined by US. Older patients have more and more calcified cartilage, also in the ventral part.

Every pathological finding should always be displayed in at least two planes if possible. Furthermore, in the case of extensive changes, the entire pathology should be displayed using panoramic imaging or a short video if this is technically possible. Color Doppler sonography (CDS) and, in selected cases, contrast-enhanced ultrasound (CEUS) should be used to improve the characterization of abnormalities. The ultrasound-guided biopsy is the standard procedure for the evaluation of masses of the chest wall.

### 1.2. Contrast-Enhanced Ultrasound of the Chest Wall: Examination Technique

The CEUS examination is dependent on the clinical context and suspected findings. Before the contrast examination, the best position of the patient should be determined using B-mode US, and the target lesion should be identified. Linear probes with high frequencies are preferred in most cases, although curved arrays with lower frequencies may be useful in cases with deep lesions. The contrast agent is administered via avenous access as a bolus injection with a dose of usually 2.4 mL of SonoVue (Bracco, Milan). The lesion should be observed continuously and recorded by a clip for at least the first 30 s after contrast administration. Thereafter, the perfusion pattern of the lesions should be examined continuously by several short examinations at 30 s intervals up to 3 min.

The chest wall lesions show a systemic vascularization pattern. On CEUS, the presence of homogeneity, the extent of enhancement, and the decrease in the enhancement can be evaluated. Furthermore, the necrotic part of the tumor is visible with CEUS and can be avoided during the biopsy.

### 1.3. Indications

Ultrasound examination of the chest wall is usually performed as a so-called “point-of-care examination”. Indications for sonography of the chest wall include:

- Localized pain of the chest wall;
- Swelling of the chest wall;
- Abnormal palpation findings;
- Targeted clarification of unclear findings of other examinations (e.g., scintigraphy, computed tomography [CT]);
- Ultrasound-guided biopsies.

### 1.4. Examination of Localized Fluid Collections of the Chest Wall

Localized fluid collections of the chest wall are rare, and the differential diagnosis is usually simple and rapid with the combination of clinical and medical history. In this context, hematomas of the chest wall, postoperative seromas, and abscesses should be mentioned in particular. Also, a cancer or metastasis can produce a localized fluid collection. On CEUS, the fluid collections do not show an enhancement.

#### 1.4.1. Hematomas of the Chest Wall

**Figure 1:**
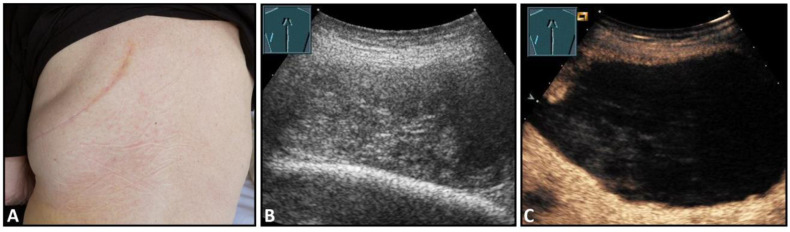
A patient after surgery for a bronchial carcinoma with a palpable chest wall tumor (**A**). B-mode US reveals a heterogeneous echogenic mass (**B**) without enhancement on CEUS (**C**), consistent with hematoma of the chest wall.

Hematomas of the chest wall are usually the result of blunt or penetrating trauma or arise after thoracic surgery. Spontaneous hematomas are found in rare cases in anticoagulated patients or in patients with coagulopathies and/or malignant tumors of the chest wall. The sonographic image depends mainly on the age or degree of organization of the hematoma. Whereas fresh hematomas are inhomogeneously hyperechoic due to their high erythrocyte content, organized hematomas are usually inhomogeneously hypoechoic or even nearly anechoic. CEUS is helpful in the differentiation of solid tumors (Figure 1).

#### 1.4.2. Postoperative Seromas

**Figure 2:**
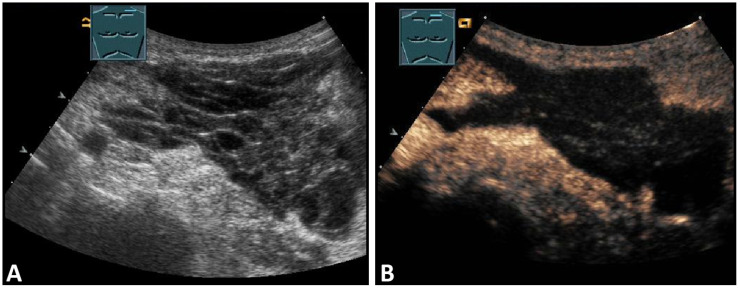
A patient after supraclavicular lymph node resection with postoperative presentation of a polyseptated mass in B-Mode US (**A**) with absent enhancement on CEUS (**B**), consistent with seroma in the upper thoracic aperture.

Seromas comprise localized collections of blood plasma or lymphatic fluid in a newly formed cavity in the area of closed surgical wounds or are the residuals of hematomas. Due to their low cellular content, seromas present on sonography as mostly anechoic structures without a distinct capsule, possibly with septa (Figure 2).

#### 1.4.3. Abscesses of the Chest Wall

**Figure 3:**
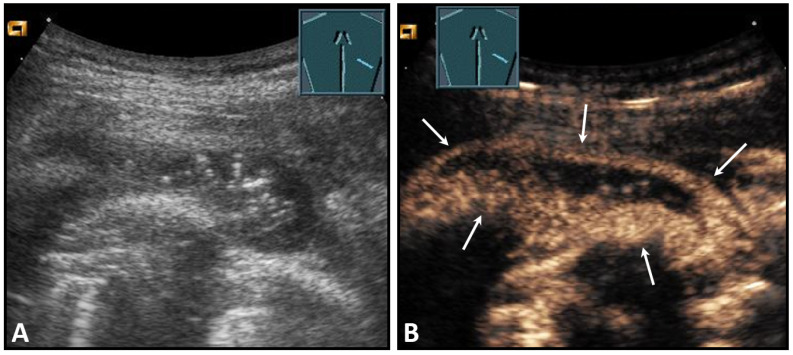
A patient with suspected granulomatous lung disease and a thoracotomy performed for histologic confirmation 7 days prior to US examination. Detection of a complex mass with gas containment on B-mode US (**A**). On CEUS, only enhancement of the capsule is observed (**B**), consistent with a cytologically confirmed abscess of the chest wall. The arrows mark the abscess capsule.

The suspicion of a chest wall abscess usually arises from the clinical presentation of the patient (swelling, redness, pain), and the role of ultrasound is to determine the extent of the process and its relation to adjacent structures. Depending on their stage, abscesses can have a very variable sonomorphology. The spectrum here ranges from mostly anechoic fluid collections with or without internal echoes to markedly inhomogeneous, anechoic structures that exhibit floating internal echoes under mild compression. An important feature of abscesses is their encapsulation, the detection of which allows the differentiation of abscesses and hematomas. On CEUS, the abscess capsule shows a marked enhancement (Figure 3).

### 1.5. Evaluation of Fractures

Patients with suspected rib fractures present with localized pain of the chest wall. Rib fractures are the most common injuries following blunt chest trauma, with an incidence of 50% in blunt thoracic trauma [2,3]. Detection of nondisplaced rib injuries is important to explain the patient’s pain. In addition, much more important than the diagnosis of the rib fracture itself is the confirmation or exclusion of associated injuries, such as pneumothorax, hemothorax, lung contusion or even hematoma of parenchymatous upper abdominal organs.

Several comparative studies have demonstrated that US is an excellent method for detecting rib fractures [4,5,6,7,8]. In a systematic review, Battle and colleagues demonstrated that US is superior to chest radiography in the detection of rib fractures [7]. In a small series, Turk et al. were able to demonstrate rib fractures using US in 18 of 20 patients who were not diagnosed by X-ray [6]. Similar results were obtained by Griffith et al., who showed that, in 50 patients, the combination of chest X-ray and oblique rib view was capable of diagnosing only 10% of rib fractures detected by US (8 of 83 fractures) [5]. In a cohort of 201 patients with blunt thoracic trauma reported by Hwang and Lee, X-ray and US results matched in only 38%. In 62% of cases, fractures were detected on US that were not previously described radiologically, or more fractures were detected on US than on X-ray in the same patients [8]. However, this is contrasted by a study by Hurley et al. in which US was only slightly better at detecting rib fractures than the combination of chest X-ray and an oblique rib view in 14 patients [9]. The average time expenditure of 13 min for the US examination was critically discussed in this study [9].

**Figure 4:**
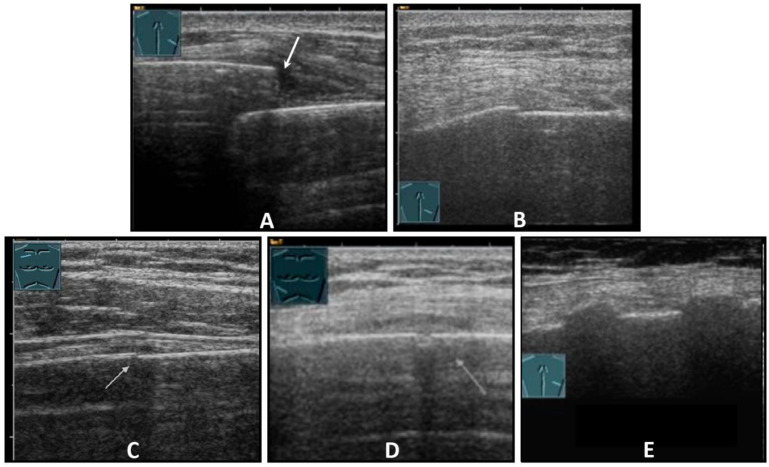
Illustration of different patterns of traumatic rib fractures: (**A**) major step formation with hematoma (arrow); (**B**) minor step formation; (**C**) minimal contour disruption (arrow) with small hematoma; (**D**) small impression (arrow) with hematoma; and (**E**) long-standing fractures with bone consolidation.

Fractures of ribs or the sternum can be diagnosed by visualizing an interruption of the cortical bone contour. Frequently, a step formation with a surrounding hematoma is found, which is visualized as a blurred-bounded, inhomogeneous, hypoechoic structure (Figure 4). Imaging of reparative processes and contusions is possible. In principle, traumatic fractures cannot be differentiated from pathological fractures unless a tumor soft tissue mantle can be visualized.

Caution should be applied when diagnosing rib fractures in the region of the costochondral joint, because a small step is often found here that can look very similar to a fracture. The surrounding hematoma can help to differentiate a fracture from a costochondral joint. The diagnosis of sternal fractures is difficult on US, as cortical interruptions in the area of synchondrosis between the corpus and the manubrium sterni as well as incomplete fusions of the sternal bone spurs can also mimic fractures [10,11]. With knowledge of these synchondrosis a sternal fracture can be diagnosed with ultrasound much easier than with normal X-ray. It should be pointed out that in the case of rib fractures, the underlying structures should be visualized for confirmation or exclusion of associated injuries.

### 1.6. Solid Masses of the Chest Wall

Tumors of the chest wall form a heterogeneous group of diseases that can originate from either soft tissue or bone (Table 1).

Primary tumors of the chest wall are rare and have an incidence of less than 2% of the population [13]. Whereas benign tumors usually grow slowly and expansively and are asymptomatic, malignant tumors of the chest wall infiltrate the surrounding structures and manifest through pain [14]. The most common malignancies of the chest wall are metastases, usually found in the ribs and originating from carcinomas of the lungs, breast, prostate, or kidneys. The role of US is to confirm the suspected clinical diagnosis in clinically benign-appearing lesions, such as lipomas, fibromas, or neurogenic tumors and to enable image-guided biopsy and the evaluation of infiltration of the surrounding structures in malignancy-suspected lesions.

#### 1.6.1. Lipomas of the Chest Wall

**Figure 5:**
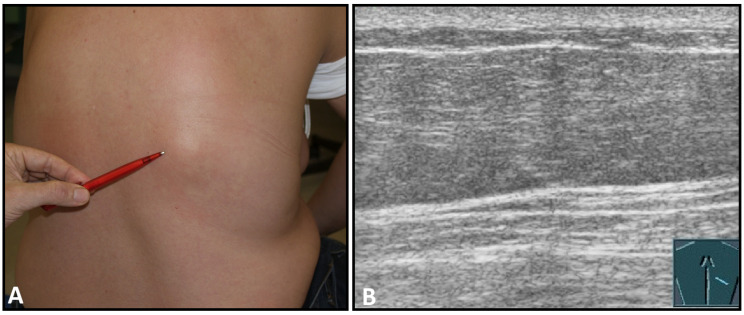
A dorsal chest wall mass with a soft echogenic subcutaneous mass consistent with lipoma (**A**). The echotexture of the lipoma is similar to that of the surrounding adipose tissue (**B**).

The most common tumors of the chest wall are lipomas, which manifest clinically as a painless and soft swelling, preferentially in adipose patients, usually between the ages of 50 and 70 years [15]. The sonomorphology is variable. In most cases (28–60%), lipomas present as a sharply localized, poorly vascularized, soft mass with an echo texture that is nearly indistinguishable from the surrounding adipose tissue. However, lipomas may also be more echoic (20–52%) or less echoic than the surrounding adipose tissue (20%) (Figure 5) [16,17].

#### 1.6.2. Chondropathia Tuberosa Costosternalis (Tietze Syndrome)

**Figure 6:**
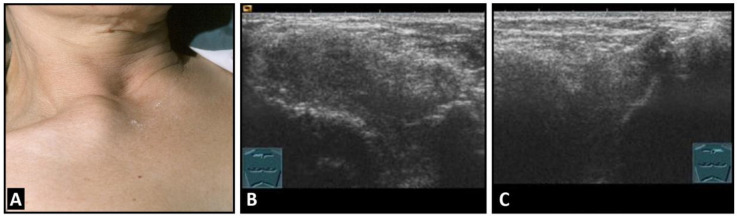
A patient with painful swelling of the right sternoclavicular joint (**A**). B-mode US shows a hypoechoic swelling similar to that in Tietze syndrome (**B**) in comparison with the contralateral left joint (**C**).

The idiopathic chondropathia of the costal cartilages at the base of the sternum is associated with pain and swelling and presents sonographically as a hypoechoic swelling of the sternoclavicular or costosternal joint with hypervascularization in power Doppler. The primary importance of this benign pathology is the differentiation from malignant infiltration (Figure 6).

#### 1.6.3. Benign Nerve Sheath Tumors

**Figure 7:**
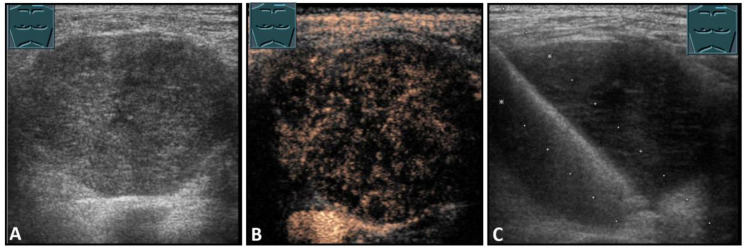
Hypoechoic tumor formation visualized by B-mode US (**A**) with low perfusion on CEUS (**B**), consistent with histologically confirmed schwannoma (**C**).

Schwannomas and neurinomas are benign neurogenic tumors originating from the nerve sheaths. Whereas neurofibromas grow along the nerve axis, schwannomas usually show eccentric growth [16]. In the thorax, both tumors originate predominantly from spinal nerve roots or intercostal nerves and usually manifest between the ages of 20 and 50 years [15]. Sonographically, neurogenic tumors appear as sharply demarcated, hypoechoic masses that may have posterior acoustic enhancement [16]. Large neurogenic tumors often show focal anechoic areas consistent with degeneration or hemorrhage [16]. An important diagnostic criterion for a neurogenic tumor is the direct detection of the associated nerve. For differentiation from malignant tumors, histological confirmation is often required (Figure 7).

#### 1.6.4. Malignant Primary Soft Tissue Tumors

**Figure 8:**
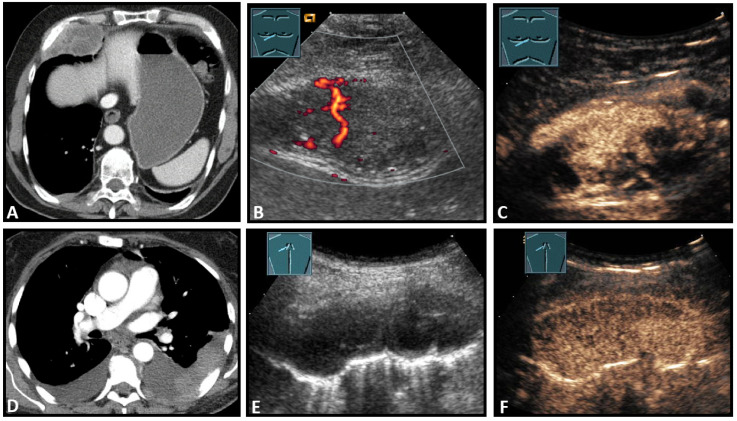
Top row: a neuroendocrine tumor on computed tomography (CT) (provided by Prof. Dr. Andreas H. Mahnken, Marburg, Germany) (**A**), CDS (**B**), and CEUS (**C**), here with inhomogeneous enhancement. Bottom row: a plasmocytoma infiltration on CT (**D**), B-Mode US (**E**), and CEUS (**F**), here with homogeneous marked enhancement.

Malignant tumors of the chest wall are characterized by infiltration of tumor tissue into the surrounding structures. On B-mode US, malignant soft tissue tumors usually present as inhomogeneous hypoechoic lesions with anechoic (necrotic) areas or calcifications and often show increased vascularization (Figure 8) [17]. The diagnosis is made histologically.

#### 1.6.5. Secondary Malignant Chest Wall Lesions

**Figure 9:**
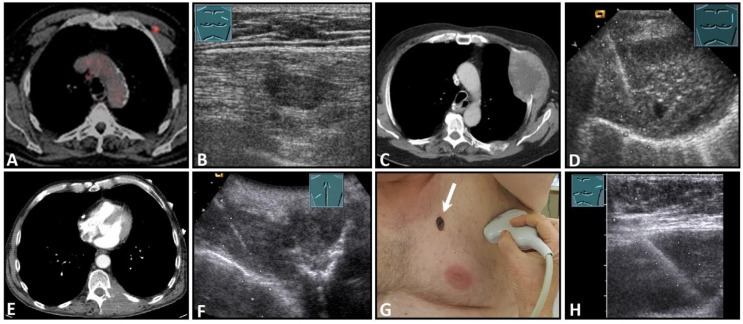
Imaging of four patients with secondary malignant chest wall lesions. Positron emission tomography computed tomography (**A**) (provided by Prof. Dr. med. Markus Luster, Marburg, Germany) in a patient with small cell lung cancer and focal lesion on B-mode US in the chest wall (**B**). A patient with metastasis of an esophageal carcinoma: imaging on CT (provided by Prof. Dr. Andreas H. Mahnken, Marburg, Germany) (**C**) and B-Mode US with visualization of the needle reflex on US-guided biopsy (**D**). Paravertebral metastasis of vocal fold carcinoma with imaging on CT (**E**) and B-Mode US with visualization of the needle reflex on US-guided biopsy (**F**). Imaging of malignant melanoma in the pectoral skin region (arrow) (**G**) with regional lymph node histologically confirmed as melanoma metastasis (**H**).

US is of high importance in the detection of malignant lesions due to its location in the near-field region. In addition to imaging, US-guided biopsy is an essential pillar of diagnostics (Figure 9).

**Figure 10:**
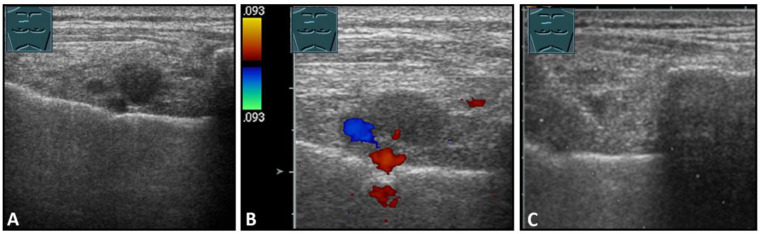
Visualization of lymph node metastases parasternal to breast carcinoma in B-mode US (**A**) and CDS (**B**), with histologic confirmation (**C**).

The lymph node region along the parasternal mammary vessels must be explored conscientiously (Figure 10).

#### 1.6.6. Osteolytic Metastases

**Figure 11:**
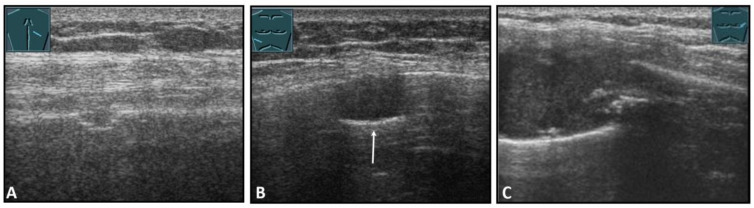
Illustration of different patterns of malignant pathologic rib pathology/rib fractures: (**A**) minor rib impression in non-small cell lung cancer; (**B**) complete bony destruction of a rib with visualization of the pleural reflex band in thymic carcinoma metastasis (arrow); and (**C**) pathologic fracture with a solid soft tissue component in melanoma metastasis.

Osteolytic metastases show an irregular interruption of the contour of the cortical bone on ultrasound, with varying degrees of inhomogeneously hypoechoic soft tissue. Destruction of the bone results in pathological sound transmission (Figure 11).

US is of considerable clinical importance in the evaluation of unclear scintigraphic findings in patients with known malignancies because it can often clearly distinguish bone fractures from metastases [18]. In unclear cases, the diagnosis of a suspicious lesion with a soft tissue component can be confirmed by US-guided biopsy.

### 1.7. Potential Limitations and Advantages of Ultrasound Compared with Cross-Sectional Imaging Methods

US examination of the chest wall has several diagnostic limitations. The US examination is generally characterized by high interobserver and interequipment variability. Furthermore, it is limited by technical difficulties, for example, in detecting the posterior location of fractures and the penetration of soft tissues in patients with excess adipose tissue or patients with large breasts [19].

Compared with US, CT and MRI provide a better overview for the detection of pathologies in the chest wall. Furthermore, although pain during the examination may be helpful in detecting the focal pathology, patient-reported pain during the US examination may limit the examination [19]. In addition, cross-sectional imaging methods such as CT and MRI provide a better overview compared with US.

Nevertheless, due to advantages such as dynamic imaging capabilities and relative simplicity, US can be used especially as a point-of-care procedure in the detection of traumatic pathologies and has a good diagnostic accuracy compared with CT as the gold standard [19,20] (Table 2).

In assessing the malignancy of chest wall neoplasia, the domain of US is in the evaluation of secondary infiltration of the chest wall by intrathoracic tumors. Here, US has a high diagnostic accuracy due to its high local resolution (Table 3). Another area of utility is the US-guided biopsy of neoplasms of the chest wall. Here, CEUS is invaluable in delineating lesional borders and in the differentiation of vital and avital tissues [22].

### 1.8. Conclusions

The chest wall is almost ideally suited for sonographic examination due to its localization close to the surface. Indications for sonography of the thoracic wall are primarily the clarification of swellings or palpable findings of the thoracic wall or the specific clarification of pain points in the area of the thoracic wall [26]. In addition, sonography of the thoracic wall also plays an important role in biopsy and surgical planning of tumors of the thoracic wall or space-occupying lesions of the lung infiltrating the thoracic wall. Last but not least, ultrasound also plays an important role in the clarification of lymph nodes [26].

## 2. Mediastinum

### 2.1. General Examination Technique

Sonographic examination of the prevascular mediastinum is limited by the ribs and sternum. Sonographic access to the mediastinum is via the supra- and parasternal, and occasionally also via the infrasternal, routes. The great vessels serve as guide structures because of their positional relationship to the heart at various levels. Suprasternal examination is performed with the patient in the supine position. The view into the upper mediastinum is facilitated by reclining the head, preferably with the thoracic spine padded underneath. Right and left rotation of the head is also helpful. Right- and left-sided positioning results in a displacement of the mediastinum, with displacement of the lung space, which allows an improved view of the mediastinum. The assessment is most favorable in the expiratory position [27].

### 2.2. Contrast-Enhanced Ultrasound of the Mediastinum: Examination Technique

Before the contrast examination, the best view should be determined using B-mode US, and the target lesion should be identified. Linear probes or curved probes should be selected depending on the depth of the lesion. The contrast agent is administered via avenous access as a bolus injection with a dose of usually 2.4 mL of SonoVue (Bracco, Milan). The lesion should be observed continuously and recorded by a clip for at least the first 30 s after contrast administration. Thereafter, the perfusion pattern of the lesions should be examined continuously by several short examinations at 30 s intervals up to 3 min.

The mediastinum has a systemic vascularization pattern. On CEUS, the presence of homogeneity, the extent of enhancement, and the decrease in the enhancement can be evaluated. The domains of CEUS are differentiation of cystic from solid lesions and avoidance of avital parts during biopsy.

### 2.3. Indications and Description of a Typical Situation

Transthoracic ultrasound (TUS) plays a minor role in the diagnosis and differentiation of mediastinal pathologies compared with other methods such as CT or magnetic resonance imaging [28]. The domain of TUS for primarily unexplained mediastinal masses is percutaneous US-guided biopsy of the anterior mediastinum (the narrow space between the dorsal sternal surface and the anterior surface of the pericardium, superior vena cava, and trachea) and, if sonographically visualizable, the paravertebral mediastinum (located between the anterior surface of the spine and the dorsal chest wall) [28,29,30,31].

### 2.4. Thymus

**Figure 12:**
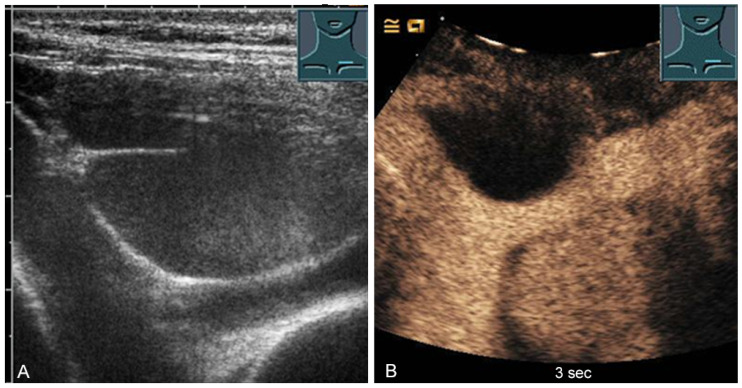
A cystic mediastinal mass on B-mode ultrasound (**A**) and contrast-enhanced ultrasound (**B**), confirmed mediastinoscopically as a regressive thymoma.

In adults, the involved thymus is mostly replaced by adipose tissue and usually cannot be visualized. Cystic involvement is possible (Figure 12) [31,32].

**Figure 13:**
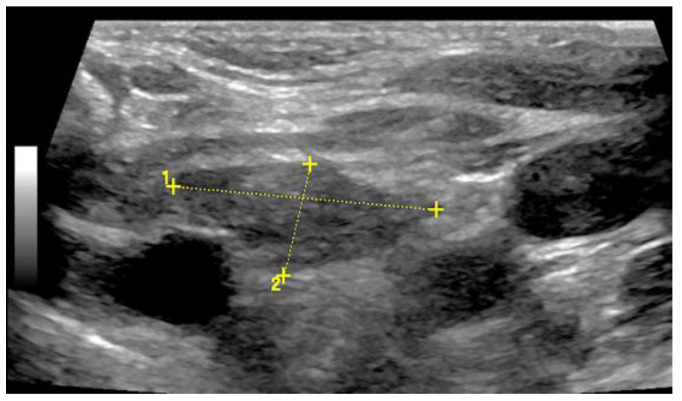
Illustration of a 2 cm × 1 cm sized thymus (thymoma) on B-mode US in an adult.

Reactive thymus enlargement (thymus rebound) can be observed after stressful situations (including surgery, burns, and chemotherapy) [31]. In childhood (2–8 years of age), thymic tissue is detectable in more than 90% of cases [33]. On US, the thymic tissue predominantly shows similar homogeneous echogenicity to that of the liver and spleen, with multiple hypoechoic strands. In individual cases, the thymic tissue shows an inhomogeneous echogenicity [34,35]. The hypoechoic strands appear as a “starry sky” on US and help to identify thymic tissue as such [34,35]. The characteristic US image is also helpful in identifying normal anatomic variants, such as cervical or retrocaval extensions of the thymus [34] (Figure 13).

### 2.5. Solid and Cystic Primary Tumors

**Figure 14:**
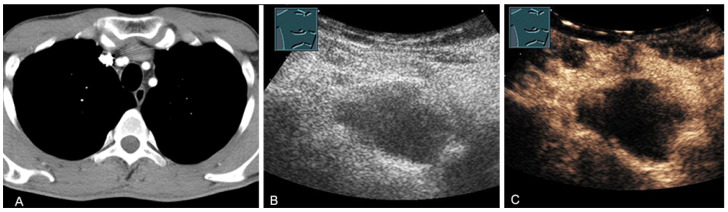
A cystic mediastinal mass on CT (provided by Prof. Dr. Andreas H. Mahnken, Marburg, Germany) (**A**), B-mode US (**B**), and CEUS (**C**), surgically confirmed as a mediastinally located bronchogenic cyst.

**Figure 15:**
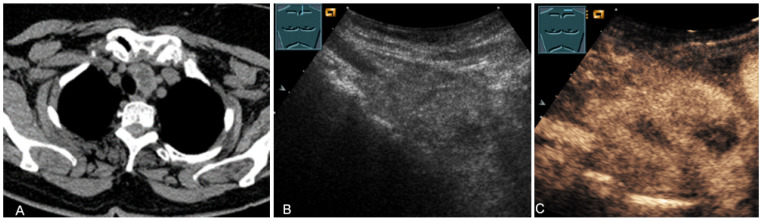
An echogenic mediastinal mass as an incidental finding on CT (provided by Prof. Dr. Andreas H. Mahnken, Marburg, Germany) (**A**), B-mode US (**B**), and CEUS (**C**), evaluated by imaging as a retrosternal located thyroid gland.

**Figure 16:**
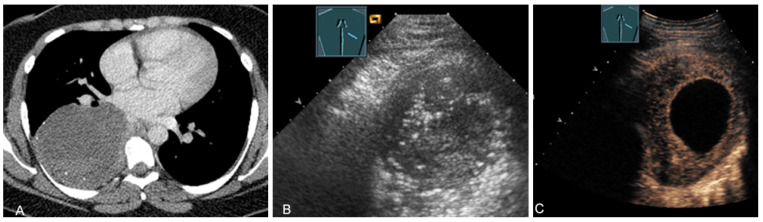
A young patient with tumor formation in the paravertebral mediastinum on CT (provided by Prof. Dr. Andreas H. Mahnken, Marburg, Germany) (**A**), B-mode US (**B**), and CEUS (**C**), surgically confirmed as ganglioneuroma.

**Figure 17:**
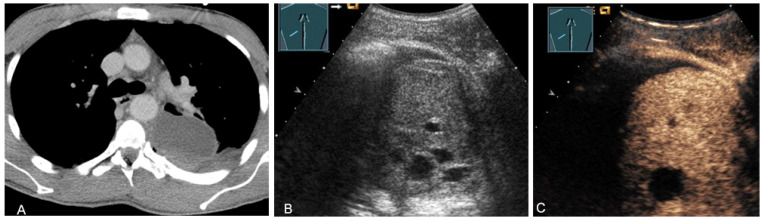
A young patient with tumor formation in the paravertebral mediastinum on CT (provided by Prof. Dr. Andreas H. Mahnken, Marburg, Germany) (**A**), B-mode US (**B**), and CEUS (**C**), surgically confirmed as schwannoma.

**Figure 18:**
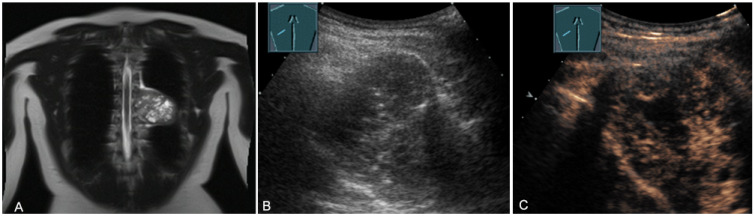
A young patient with tumor formation in the paravertebral mediastinum on magnetic resonance imaging (provided by Prof. Dr. Andreas H. Mahnken, Marburg, Germany) (**A**), B-mode US (**B**), and CEUS (**C**), surgically confirmed as a benign giant cell tumor.

The spectrum of primary mediastinal masses includes a heterogeneous group of benign and malignant pathologies [28,31,36]. Benign primary mediastinal masses include congenital cysts (Figure 14), lipomas, thyroid tissue (Figure 15), benign thymomas, thymic residues, scar tissue, ganglioneuromas (Figure 16), schwannomas (Figure 17), and giant cell tumors (Figure 18) [28,31,36].

**Figure 19:**
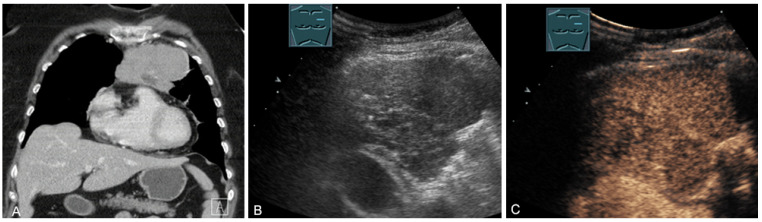
A patient with tumor formation in the anterior mediastinum on CT (provided by Prof. Dr. Andreas H. Mahnken, Marburg, Germany) (**A**), B-mode US (**B**), and CEUS (**C**), surgically confirmed as malignant thymoma stage B1.

**Figure 20:**
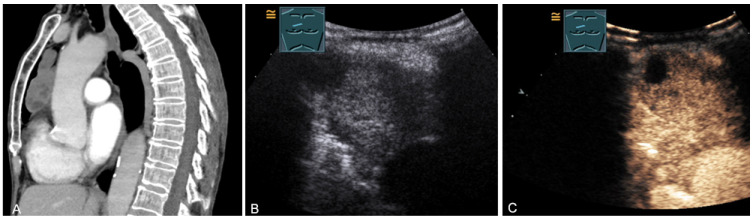
A patient with tumor formation in the anterior mediastinum on CT (provided by Prof. Dr. Andreas H. Mahnken, Marburg, Germany) (**A**), B-mode US (**B**), and CEUS (**C**), surgically confirmed as lymphoepithelioma-like malignant Thymoma B2.

**Figure 21:**
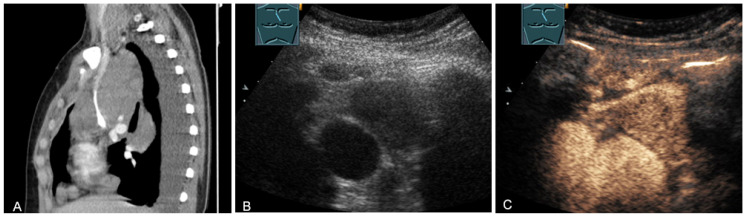
A young patient with tumor formation in the anterior mediastinum on CT (provided by Prof. Dr. Andreas H. Mahnken, Marburg, Germany) (**A**), B-mode US (**B**), and CEUS (**C**), confirmed by biopsy as Hodgkin’s disease.

**Figure 22:**
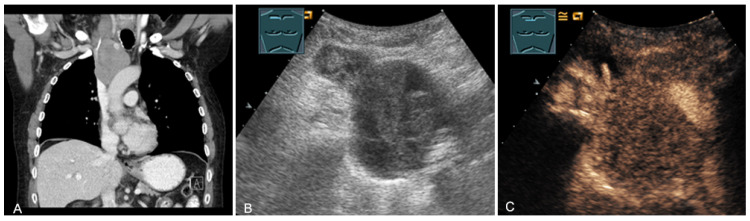
A patient with tumor formation in the anterior mediastinum on CT (provided by Prof. Dr. Andreas H. Mahnken, Marburg, Germany) (**A**), B-mode US (**B**), and CEUS (**C**), confirmed by biopsy as diffuse large B-cell non-Hodgkin’s lymphoma.

**Figure 23:**
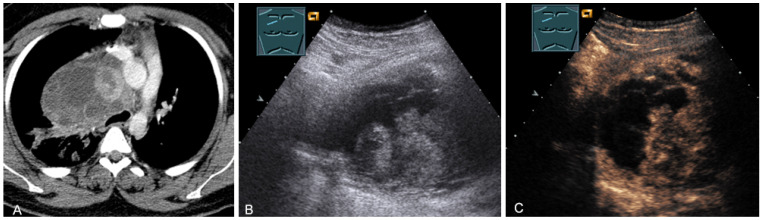
A patient, with a history of germ cell tumor, with tumor formation in the anterior mediastinum on CT (provided by Prof. Dr. Andreas H. Mahnken, Marburg, Germany) (**A**), B-mode US (**B**), and CEUS (**C**), confirmed by biopsy as mediastinal metastasis of malignant teratoma.

The last-mentioned entities are predominantly located in the paravertebral mediastinum. Primary malignant mediastinal masses of the prevascular mediastinum include malignant thymomas (Figure 19) or thymic carcinomas (Figure 20), Hodgkin’s disease (Figure 21), malignant non-Hodgkin’s lymphoma (Figure 22), and extragonadal germ cell tumors, including malignant teratomas (Figure 23) and seminoma [28,31,36].

**Figure 24:**
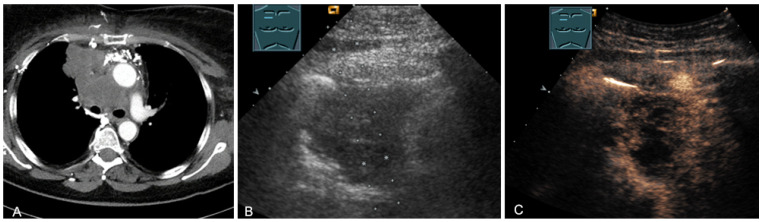
A patient with tumor formation in the anterior and middle mediastinum on CT (provided by Prof. Dr. Andreas H. Mahnken, Marburg, Germany) (**A**), B-mode US (**B**), and CEUS (**C**), confirmed by biopsy as primary bronchial carcinoma.

Malignant tumors of the thymus may have irregular borders with infiltrative growth, chaotic vascularization, cystic areas, or necrotic components on B-mode US, CDS, and CEUS [28,36,37]. These features can be used to evaluate the malignancy of the masses in the mediastinum. However, the definitive diagnosis is basically made by histological confirmation. More frequently, primary central bronchial carcinomas grow infiltrating directly into the anterior mediastinum and can be sonographically visualized in the case of pleural contact and, therefore, can also undergo US-guided biopsy (Figure 24).

**Figure 25:**
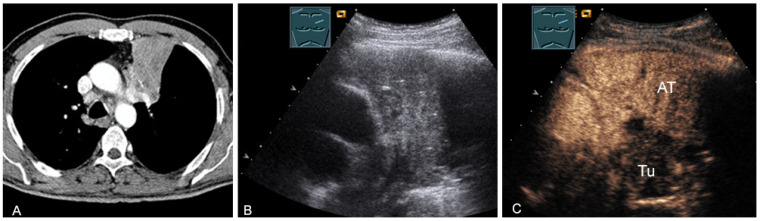
A patient with mediastinal mass on CT (provided by Prof. Dr. Andreas H. Mahnken, Marburg, Germany) (**A**), B-mode US (**B**), and CEUS (**C**), differentiating bronchial carcinoma (TU; hypoenhancing) from downstream atelectasis (AT, marked enhancement).

Central bronchial carcinomas can also be sonographically visualized by using the associated atelectasis as an “acoustic window” into the mediastinal processes [38] (Figure 25).

### 2.6. Lymphadenopathy

Mediastinal lymphadenopathy can be benign or malignant [39]. In the majority of cases, benign lymphadenopathy is associated with pulmonary diseases, including tuberculosis, sarcoidosis, histoplasmosis, anthracosis, and silicosis [39]. The most common causes of malignant lymphadenopathy are malignant lymphomas on the one hand and lymph node metastases of carcinomas or sarcomas on the other [31,39]. Endoscopic sonography (endobronchial ultrasound (EBUS) and endo esophageal endoscopic ultrasound (EUS)) is of particular importance for detection, localization, characterization, and cytological/histological confirmation [40].

#### 2.6.1. Malignant Lymphomas

In malignant lymphomas, lymph nodes present as hypoechoic in B-mode US and are often fused together forming bulky lesions [28,37]. The enlarged lymph nodes may displace or wrap around mediastinal structures [28,37]. Occasionally, a large space-occupying lymphoma manifestation (as well as central lung carcinoma) may be associated with parasternal infiltration of the chest wall, superior vena cava compression in the form of superior vena cava syndrome, and/or pleural effusion-including chylothorax [28,37]. Percutaneous US-guided biopsy has >90% accuracy for the evaluation of thoracic masses [41]. It is preferable to CT-guided biopsy whenever possible. US-guided biopsy with coarse needles (≥1 mm inner diameter) is superior to fine-needle aspiration in cases of suspected lymphoma [28,37].

#### 2.6.2. Lymph Node Metastases

**Figure 26:**
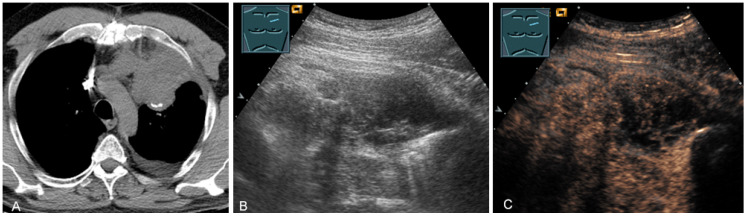
A patient with tumor formation in the anterior mediastinum on CT (**A**), B-mode US (**B**), and CEUS (**C**), with anamnestic osteosarcoma, confirmed by biopsy as mediastinal metastasis.

**Figure 27:**
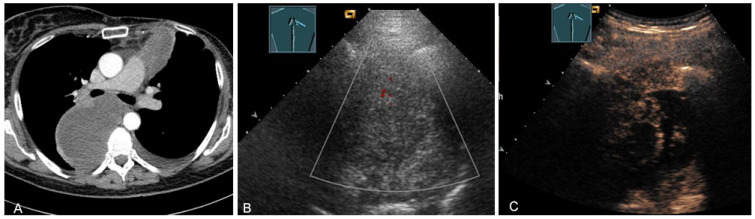
A patient with tumor formation in the anterior and paravertebral mediastinum on CT (provided by Prof. Dr. Andreas H. Mahnken, Marburg, Germany) (**A**), B-mode US (**B**), and CEUS (**C**), with anamnestic phyllodes tumor of the breast, confirmed by biopsy as mediastinal metastasis.

Enlarged lymph node metastases can be visualized on TUS if they are located in the prevascular superior mediastinum (Figure 26 and Figure 27) and inferior jugular region [28].

In bronchial carcinoma, the detection and histologic diagnosis of cervical and retrosternal–mediastinal enlarged lymph nodes are of major importance because they influence the staging and treatment of the disease. Detection of cervical lymph node metastases corresponds to an N3 manifestation and qualifies the patient for a palliative therapeutic approach [28,42]. According to Prosch et al. [42], supraclavicular lymph nodes with a transverse diameter of ≥5 mm are considered to be suspicious for malignancy and should be evaluated cytologically and histologically. Fundamentally, different underlying disease patterns do not show a specific sonographic appearance. Percutaneous US-guided biopsy of enlarged lymph nodes is therefore indicated.

### 2.7. Trachea

**Figure 28:**
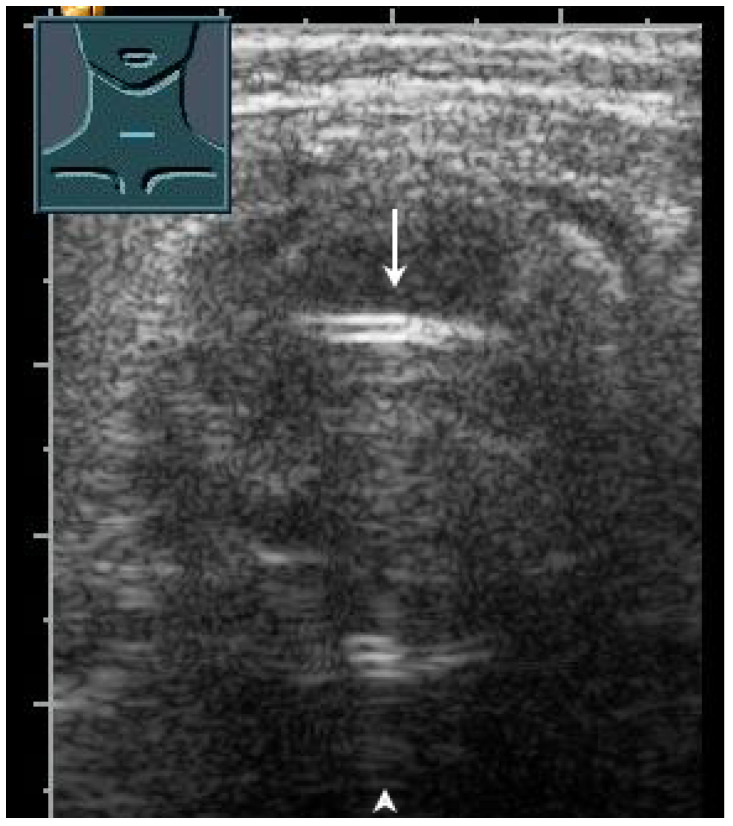
Demonstration of correct tracheal intubation. Only one air–mucosa interface within the lumen of the trachea (arrow) with comet-tail artifacts (arrowhead) and dorsal sound extinction is visualized.

US has proven to be a helpful method for the rapid evaluation of the trachea in the operating room, intensive care unit, and emergency department [43]. Various clinical applications include performing a percutaneous dilatation tracheostomy, detecting subglottic stenosis, predicting difficult intubation and stridor after extubation, determining pediatric endotracheal tube size, and verifying endotracheal tube location [43]. In a prospective study, to confirm the localization of the endotracheal tube after emergency intubation, US showed a diagnostic accuracy of 98.2% and can therefore be considered to be a reliable method [44]. These data were recently confirmed in a meta-analysis [45]. In correct tracheal intubation, only one hyperechoic air–mucosa interface with comet-tail artifacts and dorsal sound extinction is visualized (Figure 28). Esophageal intubation shows two hyperechoic air–mucosa interfaces with comet-tail artifacts and dorsal sound extinctions [44].

### 2.8. Potential Limitations and Advantages of Ultrasound Compared with Cross-Sectional Imaging Methods

The use of transthoracic mediastinal sonography is limited by several factors. These include the extensive experience required of the examiner and the limited examination possibilities in the presence of mediastinal distortions (such as those resulting from surgery or after irradiation), the overlay of air-containing lung tissue (especially in emphysema), and the limited reclination of the mediastinum [46]. Therefore, TUS of the mediastinum is indicated only on special request.

The diagnostic accuracy of TUS is dependent on the mediastinal compartment. In the evaluation of the supra-aortic, pericardial, prevascular, and paratracheal regions, sonography has a sensitivity of 89–100% and an accuracy nearly equal to that of computed tomography. However, in the aortopulmonary window and subcarinal regions, sonography has a sensitivity of only 69–81% [26,47]. Table 4 presents the diagnostic accuracy of TUS in the detection of mediastinal tumors depending on the location and is compared with CT as the gold standard.

In the case of lesions visualizable on B-mode US, CEUS can also be used for the evaluation of the malignancy. Table 5 shows the diagnostic accuracy of CEUS compared with contrast-enhanced CT and contrast-enhanced MRI.

### 2.9. Conclusions

In cases of suspected lung carcinoma, CT is recommended to evaluate mediastinal tumor formation, and, in an appropriate clinical context, complementary positron emission tomography (PET)-CT is recommended [49]. PET-CT is also recommended in cases of suspected mediastinal Hodgkin’s lymphoma [50]. Additive procedures, such as endoscopic sonography and mediastinoscopy may be used if the clinical question is relevant [31]. TUS is only indicated additively in cases of insufficient significance of the CT for the evaluation of the mediastinum or the mediastinal organs [49] and can be used independently of the specific suspected diagnosis for the histologic clarification of a mediastinal mass, especially in the presence of vena cava syndrome [51].

## 3. Diaphragm

### 3.1. General Examination Technique

**Figure 29:**
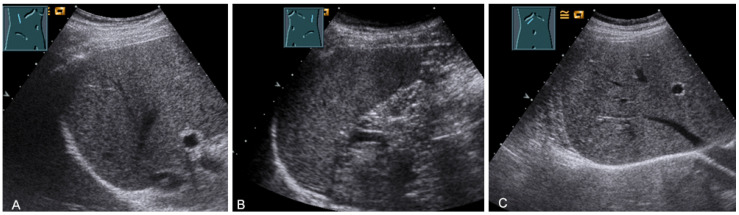
Visualization of the right-sided (**A**) and left-sided diaphragm (**B**) with the lateral intercostal view and of the right-sided diaphragm with the subcostal view (**C**).

For sonographic evaluation of the diaphragm, the patient should be in the supine position with the head angled at 30°, as this position reduces examiner variability, increases reproducibility, and enhances excursion [52,53]. The supine position increases any paradoxical movement and at the same time limits any compensatory active expiration through the anterior abdominal wall that may mask paralysis [52]. From lateral intercostal approaches, the right diaphragm should be examined through the liver window and the left diaphragm through the spleen window (Figure 29) [53]. Limitations of left diaphragm visualization due to the small acoustic window can be reduced by adopting a more coronal view, parallel to the ribs. Pathologic conditions such as splenomegaly or hepatomegaly with a large left lobe facilitate the evaluation of the left diaphragm [52]. In addition, the right-sided diaphragm can be visualized in the subcostal transhepatic section with cranial tilt of the transducer (Figure 29).

**Figure 30:**
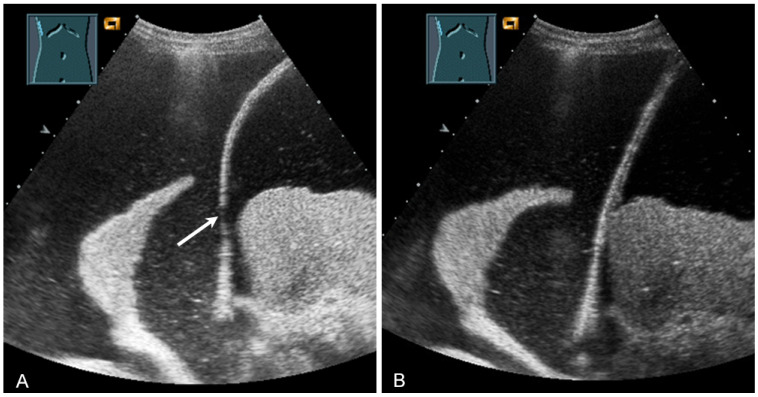
Illustration of artifact-related apparent diaphragmatic rupture (**A**, arrow) with regular findings and slightly lateral sound propagation to the diaphragm (**B**).

Border shadow artifacts may indicate an apparent diaphragmatic gap, which disappears when the transducer position is changed (Figure 30).

### 3.2. Indications and Description of a Typical Situation

Clinical indications for diaphragmatic US include characterization of diaphragmatic morphology in terms of diaphragmatic thickness and focal pathology. Diaphragmatic function must be assessed for the diagnosis and follow-up of patients with diaphragmatic paresis or diaphragmatic dysfunction. In addition, US is used to assess diaphragmatic function and morphology in ventilated patients with difficult weaning [52,53,54,55,56].

### 3.3. Examination Parameters

#### 3.3.1. Diaphragm Thickness

To measure diaphragmatic thickness, a linear transducer (≥7 MHz) should be positioned at the anterior axillary line between the 8th and 11th ribs. Visualization should be performed inferior to the costodiaphragmatic angle, where the diaphragm is attached to the inside of the chest wall (costal arch) [52,53]. Both B- and M-mode US can be used to measure diaphragm thickness [57]. The thickness measurement should be performed with visualization of both the pleural and peritoneal membranes with an angle of incidence of the US beam of approximately 90° [52]. The probe position should be marked on the skin to improve reproducibility. The pleural and peritoneal boundaries are not included in the thickness determination. The mean diaphragm thickness values obtained in healthy subjects are 1.9 ± 4 mm (95% confidence interval [CI] 1.7–2.0) in men and 1.4 ± 3 mm (95% CI 1.3–1.5) in women [58]. Because diaphragm thickness alone could lead to misdiagnosis in an underweight individual with a normally functioning diaphragm, the diaphragm thickness fraction (DTF) is used for interindividual comparisons [52] and is calculated from the difference in the thickness at the end of inspiration (tdi, end *inspiration*) and the thickness at the end of expiration (tdi, end *expiration*) in relation to the thickness of expiration (tde, end *expiration*) [52] (Equation (1)):

(1) $$DTF=\frac{\left(\mathrm{tdi},\mathrm{end}\;inspiration-\mathrm{tdi},\mathrm{end}\;expiration\right)}{\mathrm{tdi},\mathrm{end}\;expiration}\times 100$$

**Figure 31:**
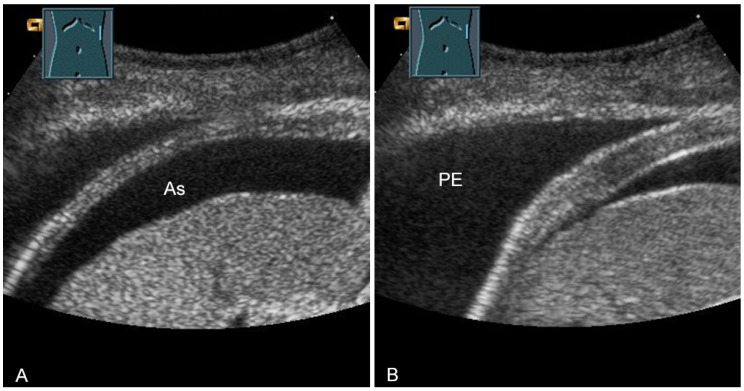
Representation of diaphragm thickness in expiration (**A**) and inspiration (**B**) in pleural effusion (PE) and ascites (As).

The thickness increase during inspiration was also used as a measure of muscle contraction. In most studies, an increase during inspiration of >20% is considered as normal [55] (Figure 31).

#### 3.3.2. Diaphragm Excursion

**Figure 32:**
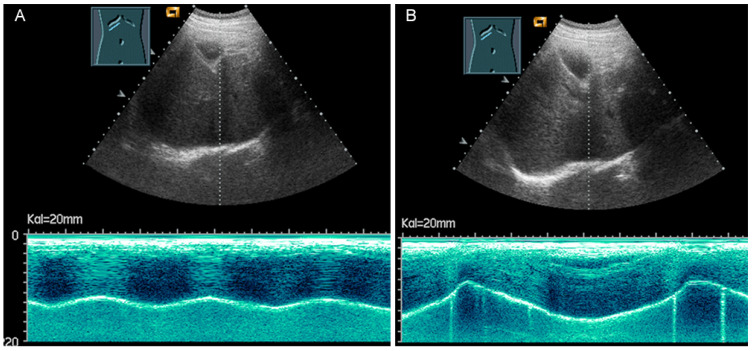
Illustration of diaphragmatic motility during quiet (**A**) and deep breathing (**B**) in a subcostal view.

Diaphragmatic excursion is measured in M-mode US with a low-frequency probe (2.5–5.0 MHz) in an anterior subcostal view through the liver window. The transducer should be positioned between the middle clavicular line and the anterior axillary line and directed medially, cranially, and dorsally to visualize the posterior third of the right diaphragm, approximately 5 cm lateral to the inferior vena cava foramen [52,53]. The amplitude (distance between the highest and the lowest points of the diaphragmatic movement) and the speed are measured in both quiet breathing and forced inspiration (the sniff maneuver) (Figure 32) [52,53]. The amplitude of diaphragmatic excursion is highly dependent on physical constitution and ranges from approximately 1–2 cm (resting breathing) to 7–9 cm (forced breathing) [59]. The diaphragm excursion can be determined only in spontaneously breathing patients.

### 3.4. Pathological Situations

#### 3.4.1. Diaphragmatic Morphology

**Figure 33:**
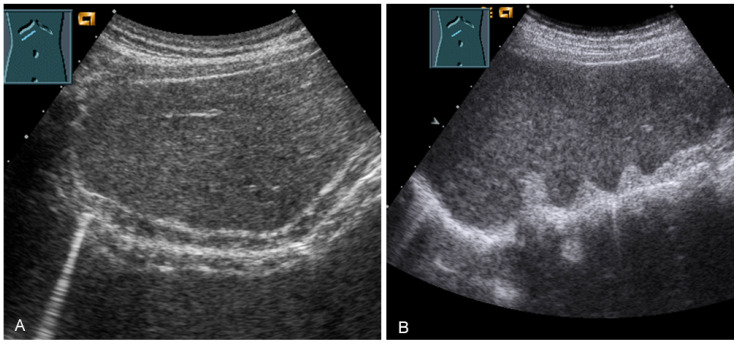
Illustration of a homogeneously thickened diaphragm (**A**) and diaphragmatic indentations (**B**) in patients with chronic obstructive pulmonary disease.

**Figure 34:**
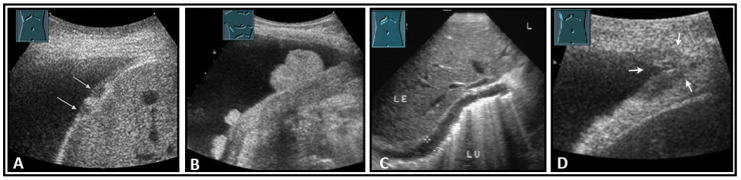
Illustration of nodular (**A**: hypoechoic flat (arrow); **B**: hyperechoic polypoid) and flat diaphragmatic metastases (**C**, between markers) and a metastasis (arrow) in the diaphragmatic rib angle (**D**). LE: liver; LU: lung.

The diaphragm presents as a three-layered structure with external bounding pleural and peritoneal membranes and a muscular layer [60]. The diaphragm thickness varies depending on breathing, among other factors. Transhepatic acoustic radiation and visualization of the diaphragmatic rib angles are of particular importance. In patients with chronic obstructive pulmonary disease, diaphragmatic hypertrophy and/or diaphragmatic furrows can be demonstrated as pseudotumors (Figure 33). In tumor staging, visualization of the diaphragm should be performed to exclude or prove diaphragmatic metastases and areal and nodular lesions can be differentiated (Figure 34).

#### 3.4.2. Diaphragmatic Paresis

The benefits of US for the examination of diaphragmatic function and detection of diaphragmatic paresis or dysfunction have been described in several publications [52,55,61,62,63,64,65,66,67,68]. A chronically paralyzed diaphragm is thin and atrophic and does not increase in thickness during inspiration [52,61]. Diaphragmatic atrophy is defined as a thickness of <1.5 mm in men and <1.1 mm in women [58]. Furthermore, a DTF of <20% is compatible with paresis [52]. Diaphragmatic paresis is evident on real-time clinical orienting examination and M-mode US as the absence of excursion during quiet and deep breathing and as the absence of or paradoxical movement during the sniff maneuver [52]. In patients with sonographic follow-up, an increase in thickness during inspiration over time was associated with an improvement in inspiratory function and an increase in vital capacity [61].

#### 3.4.3. Ventilated Patients with Complicated Weaning

Mechanical ventilation is associated with decreased muscle strength and a decrease in the contractile function of the diaphragm within 48 h of intubation [61]. Bedside sonographic functional monitoring of the diaphragm is of clinical importance. A meta-analysis showed that both diaphragm excursion assessment and diaphragm thickness fraction determination are parameters that can be usefully used to predict weaning success in invasively ventilated patients, although the cut-off values vary considerably between studies [69,70]. A diaphragmatic excursion of <1 cm and a diaphragmatic thickness fraction of <30% are considered criteria for diaphragmatic dysfunction and are associated with an increased risk of weaning failure after mechanical ventilation [62,71].

#### 3.4.4. Congenital Diseases and Hernias

Lung US is considered a reliable, cost-effective, and (due to the absence of radiation exposure) safe method for use at the bedside (point-of-care) in the neonatal unit [72,73]. The diagnostic characteristics of pulmonary diseases in neonates have already been described in several publications [72,74,75,76,77,78,79].

Point-of-care lung US can provide rapid diagnosis of congenital diaphragmatic hernias and is particularly helpful in cases of absent prenatal diagnosis [74]. Congenital diaphragmatic hernia can be detected on ultrasound by the absence of a hyperechoic diaphragmatic line, absence of a pleural line and pleural sliding in the affected hemithorax, absence of A-lines in the affected area, or presence of intestinal loops or parenchymatous organs (liver, spleen) in the thorax [74].

#### 3.4.5. Other Pathological Findings

The diagnostic relevance of diaphragmatic US in patients with chronic obstructive pulmonary disease, patients with diaphragmatic dysfunction due to neuromuscular diseases, and trauma or intensive care patients has already been described [55]. For more detailed information, we refer you to the published literature [55,70,80,81,82,83,84,85,86,87,88,89,90,91,92,93,94,95,96,97,98].

### 3.5. Potential Limitations and Advantages of Ultrasound Compared with Cross-Sectional Imaging Methods

The diaphragm is not completely visible sonographically on the right and especially on the left side. Diaphragmatic US is dependent on the cooperation of the patient. Furthermore, the normal values for diaphragmatic pathologies that have been developed so far were established for the right hemidiaphragm only [53]. However, in the context of real-time examination, US represents the first imaging modality for the diagnosis of diaphragmatic paresis. Table 6 presents the diagnostic accuracy of US compared with coronal CT in the detection of diaphragm dysfunction.

In the detection of acute diaphragmatic injuries, TUS is not a suitable diagnostic method due to its low sensitivity. Table 7 presents the diagnostic accuracy of TUS compared with CT in the detection of diaphragmatic injuries.

### 3.6. Conclusions

The domains of diaphragmatic US are in the examination and assessment of diaphragmatic function in intensive care patients, patients with chronic obstructive pulmonary disease or neuromuscular disease, and neonates with congenital diaphragmatic hernias [53,57,74]. Diaphragmatic excursion is evaluated in M-mode US with a low-frequency probe (2.5–5 MHz) and can provide information on diaphragmatic dysfunction in spontaneously breathing patients [52,53,103]. Diaphragm thickness is measured with a linear transducer (≥7 MHz) in B- or M-mode and can be used to analyze muscle function and predict extubation outcome [53,57,103]. For a reliable diagnosis of hernias, overview imaging such as CT and esophagogastroduodenoscopy are the standard procedures.

## Figures and Tables

**Table 1:** Important benign and malignant tumors of the chest wall [12].

**Benign tumors**	
**Soft tissue tumors**	**Bone tumors**
Lipomas	Fibrous dysplasia
Chondropathia tuberosa (Tietze syndrome)	Osteochondromas
Fibromas	Enchondromas
Neurogenic tumors	Aneurysmal bone cysts
Desmoid tumors	Langerhans cell histiocytosis
Elastofibroa dorsi	
**Malignant tumors**	
**Soft tissue tumors**	**Bone tumors**
Liposarcomas	Chondrosarcomas
Angiosarcomas	Myelomas
Malignant fibrous histiocytomas	Osteosarcomas
Malignant peripheral nerve sheath tumors	Ewing sarcomas
Metastases	Metastases

**Table 2:** Diagnostic performance of B-mode ultrasound in traumatic pathologies compared with computed tomography as the gold standard.

Pathology	Cases	Year	Author	Sensitivity (%)	Specificity (%)
**Traumatic lesions**	17	2018	Sabri et al. [2]	65.0	95.4
**Rib fracture**	145	2021	Çelik et al. [21]	91.3	72.7

**Table 3:** Diagnostic performance of B-mode ultrasound, computed tomography, and magnetic resonance imaging for evaluating malignancy in the chest wall compared with histopathologic examinations as the gold standard.

Imaging Method	Cases	Year	Author	Sensitivity (%)	Specificity (%)
B-TUS *	136	2008	Bandi et al. [23]	89	95
B-TUS **	119	2020	Wang et al. [24]	85	84
CT *	136	2008	Bandi et al. [23]	42	100
**MRI**	31	2021	Kolta et al. [25]	96	72

* Chest wall involvement of lung cancer; ** Differentiation of local recurrences and benign masses in patients with breast cancer. B-TUS: B-mode transthoracic ultrasound; CT: computed tomography; MRI: magnetic resonance imaging.

**Table 4:** The diagnostic performance of B-mode ultrasound in the detection of mediastinal tumors compared with CT as the gold standard.

Mediastinal Compartments	Cases	Year	Author	Sensitivity (%)	Specificity (%)
**Supra-aortic**	174	1990	Wernecke et al. [47]	98.0	100.0
**Paratracheal**	168	1990	Wernecke et al. [47]	89.0	99.0
**Aorticopulmonary**	158	1990	Wernecke et al. [47]	81.0	99.0
**Prevascular**	81	1990	Wernecke et al. [47]	92.0	100.0
**Subcarinal**	75	1990	Wernecke et al. [47]	69.0	100.0
**Pericardial**	83	1990	Wernecke et al. [47]	100.0	100.0
**Posterior mediastinum**	88	1990	Wernecke et al. [47]	6.0	100.0
**Paravertebral**	88	1990	Wernecke et al. [47]	11.0	100.0

**Table 5:** Diagnostic performance of contrast-enhanced ultrasound, contrast-enhanced magnetic resonance imaging, and contrast-enhanced computed tomography for evaluating malignancy in mediastinal mass, assuming histopathological findings as the gold standard.

Imaging Method	Cases	Year	Author	Sensitivity (%)	Specificity (%)
**CEUS**	136	2020	Pan et al. [36]	66.7	80.0
**CEMRI**	136	2020	Pan et al. [36]	88.9	66.7
**CECT**	119	2018	Pandey et al. [48]	94.0	90.0

CECT: contrast-enhanced computed tomography; CEMRI: contrast-enhanced magnetic resonance imaging; CEUS: contrast-enhanced ultrasound.

**Table 6:** Diagnostic performance of B-mode transthoracic ultrasound and computed tomography for evaluating diaphragm dysfunction, assuming diaphragmatic fluoroscopy and/or clinical diagnosis as the gold standard.

Imaging Method	Cases	Year	Author	Sensitivity (%)	Specificity (%)
TUS *	66	2014	Boon et al. [99]	93	100
CT (right hemidiaphragm) **	72	2017	Sukkasem et al. [100]	100	88
CT (left hemidiaphragm) **	72	2017	Sukkasem et al. [100]	100	77

* Gold standard: clinical diagnosis taking into account chest radiographs, fluoroscopy, phrenic nerve conduction studies, diaphragm electromyogram, and/or pulmonary function tests; ** Gold standard: diaphragmatic fluoroscopy. CT: computed tomography; TUS: transthoracic ultrasound.

**Table 7:** Diagnostic performance of B-mode ultrasound and computed tomography for evaluating diaphragmatic injuries, assuming surgical evaluation as the gold standard.

Imaging Method	Cases	Year	Author	Sensitivity (%)	Specificity (%)
**TUS**	24	2019	Sharifi et al. [101]	50	100
**CT**	45	2002	Larici et al. [102]	84	77

CT: computed tomography, TUS: transthoracic ultrasound.

## Data Availability

Not applicable.
